# Schmallenberg Virus Antibodies in Adult Cows and Maternal Antibodies in Calves

**DOI:** 10.3201/eid2005.130763

**Published:** 2014-05

**Authors:** Armin R.W. Elbers, Norbert Stockhofe, Wim H.M. van der Poel

**Affiliations:** Central Veterinary Institute, Lelystad, the Netherlands

**Keywords:** Schmallenberg virus, antibodies, cows, calves, novel orthobunyavirus, biting midges, Culicoides spp., immunity, ruminants, vaccine, vaccination, viruses, the Netherlands, ruminant livestock, maternal antibodies, passive immunity

**To the Editor:** Schmallenberg virus (SBV), a novel orthobunyavirus that is transmitted by *Culicoides* spp. biting midges, spread through herds of ruminants across Europe during 2011–2013. The virus reached as far as Finland in the north, the Republic of Ireland in the west, Turkey in the east (*1*), and Spain in the south. The clinical effect of SBV infection in ruminant livestock appears to be limited (*2*), and a vaccine to prevent the infection has been developed (*3*). There are no data to refute the assumption that natural SBV infection results in long-term immunity, as was seen earlier with natural infection of cattle with bluetongue virus serotype 8 (*4*). Newborn calves acquire passive immunity by ingestion and absorption of antibodies present in colostrum. Passive immunity can, however, block the production of serum antibodies when vaccine is administered to calves that have maternally derived antibodies (*5*). To determine the titers and persistence of SBV antibodies in adult cows and the decay of maternal antibodies in calves over time, we studied a herd of cattle from a dairy farm in the eastern Netherlands during April 2012–April 2013.

The dairy farm is the only location in the Netherlands where monitoring for biting midges was continuously conducted during the 2011–2013 SBV epidemic and where SBV RNA was detected in biting midges caught during 2011–2012 (*6**,**7*). The dairy herd comprised 110 animals: 60 milking cows (average age 4.0 years) and 50 heifers (average age 1.5 years) and calves (<1.0 year of age). No clinical signs or symptoms of SBV infection were observed in any of the cattle at the end of 2011 or during 2012. However, during the study period, 3 calves were stillborn, none of which had the characteristic malformations observed after SBV infection. Gross pathology confirmed that the calves did not have SBV infection, and all tissue samples were negative for SBV by reverse transcription PCR.

During the 12-month study, we obtained 4 blood samples from all animals in the herd. A virus neutralization test (VNT) was used to test the samples for antibodies (*8*). For optimal specificity and sensitivity, the VNT cutoff dilution was set at 1:8. Test dilutions ranged from 1:4–1:512. All samples were tested in duplicate; titers were determined using the Reed-Münch method and expressed on a log_2_ scale. 

Blood samples were first obtained from the herd on April 19, 2012, after retrospective detection of SBV RNA in biting midges that had been collected from the farm on September 14, 2011 (*6*). The remaining 3 blood samples for each animal were collected on September 17, 2012; December 9, 2012; and April 23, 2013 (5, 8, and 12 months, respectively, after the first collection). SBV VNT results for the initial blood samples were positive for all cows ≥1 year of age and for all but four 6-month-old calves. One year later, blood samples for 98% of the cows ≥1 year of age and 50% of the cows <1 year of age were SBV seropositive. During the year, the mean log_2_ VNT titer of the adult cows dropped from 8.3 to 6.7.

It can be assumed that cows ≥1 year of age became infected with SBV around the time SBV-infected *Culicoides* biting midges were detected on the farm in September 2011 (*6*). Thus, at least 19 months after natural infection, these cows were probably protected against SBV when re-exposed to the virus. Of all cattle tested, 11 heifers seroconverted between April 2012 and September 2012, and 1 cow seroconverted between the September and December 2012 samplings. The low rate of seroconversion was matched by a 6× lower proportion of SBV-infected *Culicoides* midges found in 2012 than in 2011 (*7*). We also assume that the level of SBV circulating in the area during 2012 was lower than that in 2011.

Blood samples obtained from 13 calves ≤30 days after birth had a mean log_2_ SBV VNT titer of 8.0 (range 6.5–9.5) and were seronegative at the last blood sampling on April 23, 2013 (Figure). The mean length of time between birth and the first detection of seronegative status was 180 days (range 120-240). 

**Figure:**
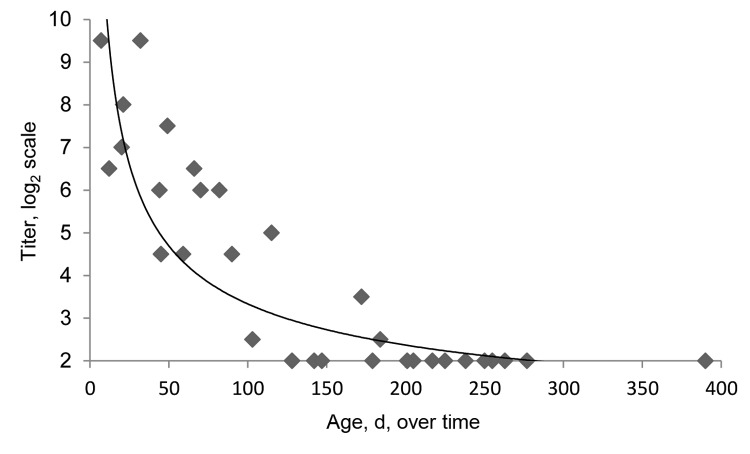
Schmallenberg virus antibody titers for 13 calves in a study to determine the decline of maternal antibodies in calves, the Netherlands, 2012–2013. Titers were determined by using a virus neutralization test and 2–4 blood samples per calf over time.

There are few reports regarding the decay of maternal antibodies against orthobunyaviruses in ruminants. Tsutsui and colleagues (*9*) showed that dairy calves lost their maternally derived antibodies against Akabane virus at ≈4 months of age, and Grimstad and colleagues (*10*) showed that young white-tailed deer lost their maternally derived antibodies against Jamestown Canyon virus at 5–6 months of age. Consistent with those findings, our results show that calves lose maternally derived SBV antibodies at ≈6 months of age and can then be effectively vaccinated against SBV.
